# Changing Molecular Epidemiology of Hepatitis A Virus Infection, United States, 1996–2019

**DOI:** 10.3201/eid2706.203036

**Published:** 2021-06

**Authors:** Sumathi Ramachandran, Guo-Liang Xia, Zoya Dimitrova, Yulin Lin, Martha Montgomery, Ryan Augustine, Saleem Kamili, Yury Khudyakov

**Affiliations:** Centers for Disease Control and Prevention, Atlanta, Georgia, USA

**Keywords:** Hepatitis A virus, genotype, surveillance, outbreaks, technical assistance, molecular surveillance, viruses, food safety, sexually transmitted infections, United States

## Abstract

Hepatitis A virus (HAV) genotype IA was most common among strains tested in US outbreak investigations and surveillance during 1996–2015. However, HAV genotype IB gained prominence during 2016–2019 person-to-person multistate outbreaks. Detection of previously uncommon strains highlights the changing molecular epidemiology of HAV infection in the United States.

Hepatitis A virus (HAV) is transmitted primarily through person-to-person contact or exposure to contaminated food or water. After the introduction of hepatitis A vaccine recommendations in the United States in 1996, reports of hepatitis A cases decreased progressively from 1999 to 2011 by a total of ≈95% (*1*,*2*). However, we recently showed that hepatitis A cases increased 294% during 2016–2018 compared with 2013–2015 among persons who use drugs (injection or noninjection), persons experiencing homelessness, or men who have sex with men (*3*,*4*). 

HAV strains infecting humans are genetically classified into genotypes I, II, and III. Genotype I is further divided into subtypes A, B, and C, and genotypes II and III are divided into subtypes A and B. In this study, we investigated HAV genotype and strain distributions in the United States during 1996–2019.

Genetic testing was performed by using DNA sequencing, and we included HAV sequences obtained from 9,203 specimens collected during outbreak investigations and surveillance activities conducted by the Centers for Disease Control and Prevention (CDC) or state health departments during 1996–2019 (Appendix Table). We performed phylogenetic analysis of a 315 base-pair fragment of the HAV viral protein 1–amino terminus of 2B genomic region amplified from serum specimens (Appendix Figure 1). 

We found that during 1996–2015, HAV genotype IA was most common among specimens collected through surveillance (93%; 1,587/1,706) and outbreak investigations (84.4%; 706/836;); genotype IB was detected among only 6.4% (110/1,706) of surveillance and 15.2% (127/836) of outbreak specimens. Genotype IIIA was detected in <0.5% of both collections (Table). During 2016–2019, a total 6,661 outbreak specimens were collected from many states across the country (Appendix Figure 2). Sequences from these outbreaks represented ≈19% of all HAV cases reported to CDC through the National Notifiable Diseases Surveillance System (*4*), ≈3 times more specimens than were collected during 1996–2015. Among the 6,661 specimens collected during 2016–2019, genotype IA was identified in 15.7% of specimens, IB in 82.8%, and IIIA in 1.5% (Table).

**Table:** Genotype distribution of HAV specimens collected through surveillance programs and outbreak investigations, United States, 1996–2019*

Year	No. (%) HAV surveillance specimens †					No. (%) HAV outbreak specimens‡					No. (%) HAV specimens			
	Total	IA	IB	IIIA		Total	IA	IB	IIIA		Total	IA	IB	IIIA
1996–2015	1,706	1,587 (93.0)	110 (6.4)	9 (0.5)		836	706 (84.4)	127 (15.2)	3 (0.4)		2,542	2,293 (90.2)	237 (9.3)	12 (0.5)
2016–2019	ND					6,661	1,046 (15.7)	5,518 (82.8)	97 (1.5)		6,661	1,046 (15.7)	5,518 (82.8)	97 (1.5)

*Since 2014, the United States has not had established HAV surveillance programs in place to provide specimens for molecular testing of surveillance cases. Specimens collected as part of outbreak investigations during 2016–2019, might include some sporadic cases. During 1996–2015, ≈130 HAV specimens/year were tested; During 2016–2019, 1,660 specimens/year were tested. HAV, hepatitis A virus; ND, no data. †Surveillance specimens tested by the Centers for Disease Control and Prevention (CDC) during 1996–2013 included sentinel county surveillance (n = 1,234) during 1996–2006; the Emerging Infectious Disease Program (n = 418) during 2007–2013; and the Border Infectious Disease Surveillance (n = 54) system during 2007–2013. ‡Outbreak specimens sequenced during 1996–2015 (n = 836) were tested by CDC. Outbreak specimens sequenced during 2016–2019 (n = 6,661) included data from CDC testing (n = 5,068) and data captured through technical assistance offered to sites, which included data from Sanger sequences reported to CDC for technical assistance and analysis from state and local public health laboratories (n = 341) and the Food and Drug Administration (n = 3); data from ultradeep sequencing and submission to the Global Hepatitis Outbreak and Surveillance Technology portal by state and local public health laboratories (n = 1,249).

Among all 9,203 tested specimens, we identified 1,055 HAV strains that we defined as having unique genetic variants; 352 (33.4%) HAV strains were identified from specimens collected during 2016–2019 (Figure). Genetic analyses demonstrate that 63.4% (n = 102) of genotype IB strains and 40% (n = 6) of genotype IIIA strains identified during 2016–2019 belonged to 2 large genetic clusters or groups of closely related HAV strains (Figure), but genotype IA strains were distributed among many small genetic clusters.

**Figure:**
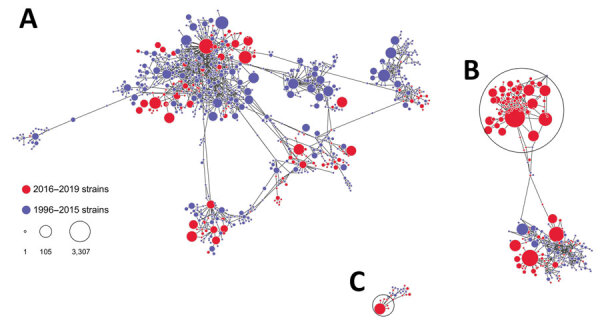
Genetic relatedness among hepatitis (HAV) strains, United States, 1996–2019. A) IA strains; B) IB strains; C) IIIA strains. Nodes represent HAV strains, and the size of node is proportional to the frequency of the strain; larger nodes denote more frequent detection. Distance between nodes approximates genetic closeness of HAV strains. Genetic clusters of closely related HAV strains encompassing a large fraction of all strains in HAV genotypes IB and IIIA are circled. Visualization created by using Gephi software (https://gephi.org).

The CDC-developed Global Hepatitis Outbreak and Surveillance Technology (GHOST) system improved molecular testing capabilities of state and local health departments during the 2016–2019 multistate outbreaks. Molecular epidemiologic methods have helped clarify HAV transmissions within networks of persons with similar risk factors (*5*). By using genetic testing, CDC has assisted in 25 outbreak investigations associated with a common source transmission by contaminated food (*6*,*7*) and person-to-person transmissions (*8*,*9*). 

For 20 years (1996–2016), during the national decrease in HAV cases attributed to increased vaccination, genotype IA was the most detected genotype. However, genotype IB cases associated with outbreaks in multiple states increased during 2016–2019. During that time, IB became the most common genotype, detected in 83% of specimens collected across many states (Appendix Figure 2). 

Findings from the National Health and Nutrition Examination Surveys during 1999–2012 revealed that despite the overall increase in HAV antibody among children, prevalence of HAV antibody among US-born adults was low (24%), indicating decreasing immunity to HAV (*10*). However, our molecular data indicate that the increase in number of HAV cases observed in outbreaks during 2016–2019 might not be attributable solely to the decline in the population’s HAV immunity. Because HAV genotype IA was dominant in the United States for years, the large person-to-person outbreaks during 2016–2019 reasonably could be expected to be caused by genotype IA strains widely circulating in the country, but our genetic analysis shows predominance of the previously rare HAV genotype IB strains. Identification of 1 large cluster and several small genetic clusters suggests >1 introduction of genotype IB to the affected population in multiple states during 2016–2019. On the basis of these findings, we hypothesize that genotype IB was introduced from regions of the world where these strains are endemic and could be responsible for initiation of the outbreaks among vulnerable populations (Appendix Figure 3). GHOST was instrumental in identifying changes in molecular epidemiology of HAV infections and is an example of novel emerging technologies that can be used for national viral hepatitis molecular surveillance program.

Our observations are hallmarks of a change in HAV molecular epidemiology in the United States. GHOST technology is improving hepatitis detection at the state and local level. Our findings emphasize the need for systematic HAV surveillance for strain characterization, timely detection of transmission clusters, and assistance in guiding public health interventions and vaccination efforts. 

Appendix Additional information on the changing molecular epidemiology of hepatitis A virus infection, United States.
