# Supplementary material for: Changing Molecular Epidemiology of Hepatitis A Virus Infection, United States, 1996–2019
Source: Emerg Infect Dis. 2021 Jun;27(6):1742–5. doi: 10.3201/eid2706.203036 (PMC8153864; doi:10.3201/eid2706.203036)
Supplement: Appendix — Additional information on the changing molecular epidemiology of hepatitis A virus infection, United States. [file 20-3036-Techapp-s1.pdf]

Article DOI: https://doi.org/10.3201/eid2706.203036

Changing Molecular Epidemiology of Hepatitis A Virus Infection, United States, 1996–2019

Appendix

Study Methodology

The study included available immunoglobulin M (IgM) anti-hepatitis A virus (HAV)–positive serum specimens (n = 1,706) collected by Centers for Disease Control and Prevention (CDC) surveillance programs during 1996–2013; 836 specimens tested by CDC from 23 outbreak investigations during 2002–2015; and 6,661 specimens associated primarily with person-to-person outbreaks in the United States during 2016–2019 (Appendix Table) (*1*,*2*). Genetic testing of specimens collected across all states was performed by using DNA sequencing and phylogenetic analysis of a 315 nt region, at position of 2897–3288 of the HAV genome (Appendix Figures 1-3). Until January 2018, only 1 sequence per specimen was obtained by using Sanger sequencing (*3*). In February 2018, CDC implemented ultradeep sequencing (UDS) for HAV as part of Global Hepatitis Outbreak and Surveillance Technology (GHOST; *4*), which resulted in sequencing multiple HAV sequence variants from each specimen. HAV genotyping was conducted by using either a single sequence obtained by Sanger sequencing or the most frequent sequence obtained from each specimen by UDS. These sequences were used to represent strains, defined as unique genetic variants, and to identify genetic clusters. Since 2017, as part of technical assistance offered by CDC, state laboratories reported 341 Sanger sequences and the U.S. Food and Drug Administration (FDA) reported 3 from food sources. UDS data from 1,249 specimens were submitted to CDC by state and local health laboratories through GHOST as part of outbreak investigations during 2018–2019.

Study Limitations

A limitation of this study is that 81.5% of the tested specimens were collected from HAV outbreak investigations that occurred during 2002–2019 and 18.5% of specimens was collected from routine surveillance activities that occurred during 1996–2013, which represented only 1% of the total reported HAV cases. Owing to the unprecedented magnitude of the 2016–2019 person-to-person outbreaks (*4*), genetic testing of specimens was not completely proportional to the number of cases reported from each state and includes a small proportion from other outbreaks and sporadic cases. Thus, the identified change in genotype predominance likely is not generalizable to all hepatitis A cases in the United States and is weighted toward the genotypes associated with the ongoing outbreaks (*4*). The study included available remnant convenient serum specimens obtained within 6 weeks of the onset of symptoms from confirmed cases that tested IgM anti-HAV–positive by the state health departments. HAV strains from deidentified IgM-positive specimens were sequenced at CDC, and independent genetic testing also was conducted by some state health departments by using the CDC-developed UDS assay and GHOST. Despite these limitations, genetic data from the available surveillance and outbreak cases are valuable to describe the changing molecular epidemiologic pattern of viral hepatitis in the United States.

References

1. Foster MA, Hofmeister MG, Kupronis BA, Lin Y, Xia GL, Yin S, et al. Increase in hepatitis A virus infections—United States, 2013–2018. MMWR Morb Mortal Wkly Rep. 2019;68:413–5. PubMed https://doi.org/10.15585/mmwr.mm6818a2

2. Centers for Disease Control and Prevention. Widespread outbreaks of hepatitis A across the United States [cited 2021 Apr 1]. https://www.cdc.gov/hepatitis/outbreaks/2017March-HepatitisA.htm

3. Nainan OV, Armstrong GL, Han XH, Williams I, Bell BP, Margolis HS. Hepatitis a molecular epidemiology in the United States, 1996–1997: sources of infection and implications of vaccination policy. J Infect Dis. 2005;191:957–63. PubMed https://doi.org/10.1086/427992

4. Association of Public Health Laboratories. Hepatitis A virus testing and resources. 2020 Feb 10 [cited 2021 Apr 1]. https://www.aphl.org/programs/infectious_disease/Documents/2018_HAV_DiagnosticUpdate.pdf

Appendix Table. Details of molecular testing of hepatitis A virus specimens collected through surveillance programs and outbreak investigations, United States, 1996–2019*

Specimen source	Study period	RNA-positive/tested specimens (%)	Unique genotypes/RNA-positive specimens (%)
Surveillance system			
Sentinel Counties Program	1996–2006	1,234/1,510 (81.72)	403/1,234 (32.66)
Binational Border Infectious Disease Program	2000–2005	54/70 (77.14)	22/54 (40.74)
Emerging Infections Program	2007–2013	418/685 (61.02)	248/418 (59.33)
Outbreak investigations*			
Outbreaks	2000–2015	836/1018 (82.12)	99/836 (11.84)
Outbreaks, person-to-person	2016–2019	6,661/7152 (93.13)	352/6,661 (5.28)
Total	1996–2019	9,203/10,435 (88.19)	1,055/7,497 (14.07)

*Specimens tested during 2000–2015 were collected from 23 outbreak investigations. Specimens tested during 2016–2019 were primarily associated with the person-to-person outbreaks; 19% of all cases reported to the Centers for Disease Control and Prevention through the National Notifiable Disease Surveillance System (*2*).

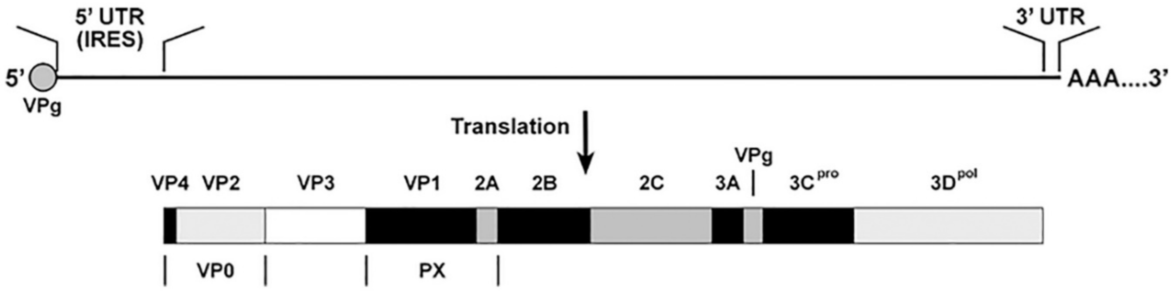

Appendix Figure 1. Hepatitis A virus (HAV) genome and viral protein 1–amino terminus of 2B (VP1–P2B) genomic region used for DNA sequencing of HAV, United States, 2015–2019. HAV was amplified from serum specimens and genetic testing was performed by using DNA sequencing. We performed phylogenetic analysis of a 315 base-pair fragment of the HAV genome and VP1–P2B region at positions 2897–3288.

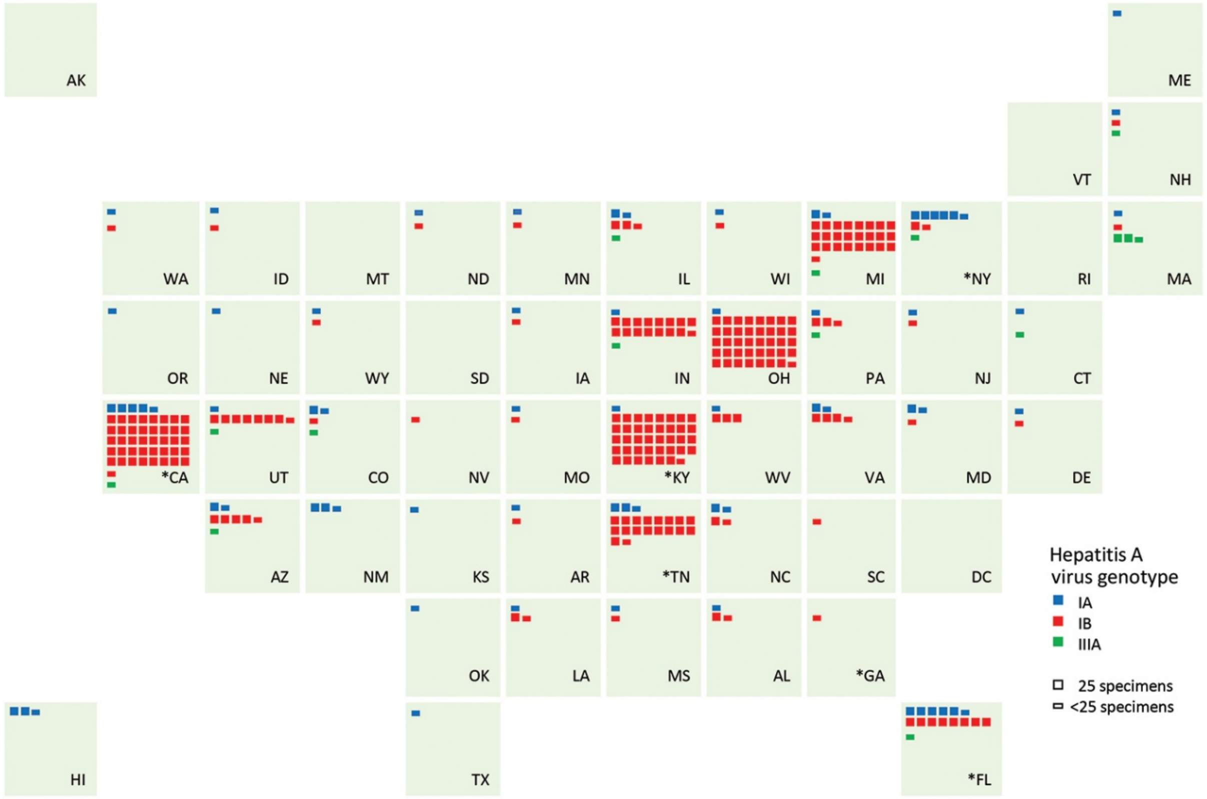

Appendix Figure 2. Capacity building and geographic distribution of hepatitis A virus (HAV) genotypes, United States, 2016–2019. In the tile grid map, each tile represents a state, arranged to represent an approximate geographic state location. Color-coded waffle charts embedded in each tile correspond to HAV genotype. The geographic distribution of HAV samples sequenced during 2016–2019 (n = 6,605) include ultradeep sequence data acquired through site testing and submitted to the Global Hepatitis Outbreak and Surveillance Technology (GHOST) portal (n = 1,249) and consensus Sanger sequence data (n = 344) reported by state health departments. Specimens without state assignment (n = 56) were not included. Asterisks indicate capacity building in state and local health departments in KY, TN, FL, NY, GA, and San Diego County, CA since 2018 that facilitated independent testing of HAV outbreaks by using GHOST. Sanger sequencing technical assistance was provided to state health laboratories in MI, CA, MN, and FL; and to laboratories of the U.S. Food and Drug Administration. AK, Alaska; AL, Alabama; AR, Arkansas; AZ, Arizona; CA, California; CO, Colorado; CT, Connecticut; DC, District of Columbia; DE, Delaware; FL, Florida; GA, Georgia; HI, Hawaii; IA, Iowa; ID, Idaho; IL, Illinois; IN, Indiana; KS, Kansas; KY, Kentucky; LA, Louisiana; MA, Massachusetts; MD, Maryland; ME, Maine; MI, Michigan; MN, Minnesota; MO, Missouri; MS, Mississippi; MT, Montana; NC, North Carolina; ND, North Dakota; NE, Nebraska; NH, New Hampshire; NJ, New Jersey; NM, New Mexico; NV, Nevada; NY, New York; OH, Ohio; OK, Oklahoma; OR, Oregon; PA, Pennsylvania; RI, Rhode Island; SC, South Carolina; SD, South Dakota; TN, Tennessee; TX, Texas; UT, Utah; VA, Virginia; VT, Vermont; WA, Washington; WI, Wisconsin; WV, West Virginia; WY, Wyoming.

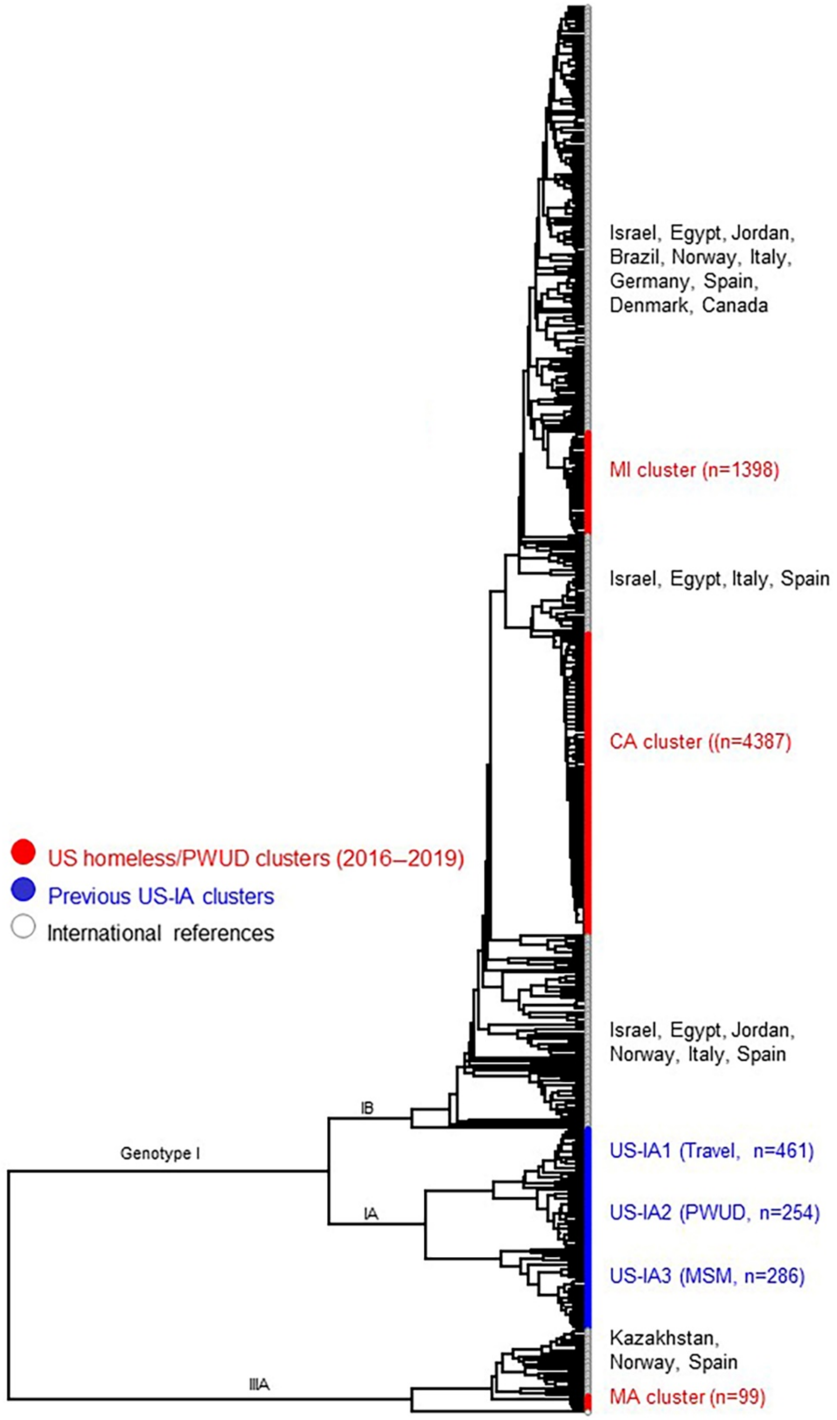

Appendix Figure 3. Hepatitis A virus (HAV) reference sequences were used to investigate HAV genotype distribution in the United States. Sequences used for the phylogenetic tree analysis were 315 bp in length from the viral protein 1–amino terminus of 2B (VP1–P2B) genomic of HAV. PWUD, persons who use drugs (injection or non-injection); MSM, men who have sex with men.
